# Synthesis, Characterization, and Photocatalytic Properties of Sulfur- and Carbon-Codoped TiO_2_ Nanoparticles

**DOI:** 10.1186/s11671-016-1353-5

**Published:** 2016-03-12

**Authors:** S. Ivanov, A. Barylyak, K. Besaha, A. Bund, Y. Bobitski, R. Wojnarowska-Nowak, I. Yaremchuk, M. Kus-Liśkiewicz

**Affiliations:** Electrochemistry and Electroplating Group, Ilmenau University of Technology, Gustav-Kirchhoff-Str. 6, 98693 Ilmenau, Germany; Department of Therapeutic Dentistry, Danylo Halytsky Lviv National Medical University, Pekarska Str. 69, 79010 Lviv, Ukraine; Department of Silicate Engineering, Lviv Polytechnic National University, S. Bandera Str. 12, 79013 Lviv, Ukraine; Department of Photonics, Lviv Polytechnic National University, S. Bandera Str. 12, 79013 Lviv, Ukraine; Faculty of Mathematics and Natural Sciences, University of Rzeszow, Pigonia Str. 1, 35959 Rzeszow, Poland; Department of Biotechnology, Biotechnology Centre for Applied and Fundamental Sciences, University of Rzeszow, Sokołowska Str. 26, 36-100 Kolbuszowa, Rzeszow, Poland

**Keywords:** TiO_2_ nanoparticle, Metatitanic acid, Thiourea, Photocatalysis, Sulfur-carbon doping, X-ray photoelectron spectroscopy

## Abstract

One-step TiO_2_ nanoparticle synthesis based on the interaction between thiourea and metatitanic acid is applied for sulfur and carbon anatase codoping. The synthesis of the doped TiO_2_ has been monitored by means of differential thermal analysis and thermogravimetric analysis (DTA-TG), which allows determining the optimal thermal conditions for the process. Electron microscopy showed micrometer-sized (5–15 μm) randomly distributed crystal aggregates, consisting of many 15–40-nm TiO_2_ nanoparticles. The obtained phase composition and chemical states of the doping elements are analyzed by means of X-ray diffraction (XRD), X-ray photoelectron spectroscopy (XPS), infrared (IR) and Raman spectroscopies, and electron paramagnetic resonance (EPR). XRD displays in both samples (doped and pristine) the existence of only one crystalline phase—the tetragonal modification of TiO_2_—anatase. Further data assessment by means of Rietveld refinement allowed detection of a slight *c* lattice parameter and volume increase related to incorporation of the doping elements. XPS demonstrated the presence of carbon and sulfur as doping elements in the material. It was confirmed that carbon is in elemental form and also present in oxygen-containing compounds, which are adsorbed on the particle surface. The binding energy for sulfur electron core shell corresponds to the established data for sulfate compounds, where sulfur is in 6+ oxidation state. The synthesized S- and C-codoped TiO_2_ showed excellent photocatalytic performance during the degradation of organic dyes (rhodamine B, methylene blue), gas-phase oxidation of ethanol under visible light, and photocatalytic hydrogen generation from ethanol under ultraviolet light.

**Electronic supplementary material:**

The online version of this article (doi:10.1186/s11671-016-1353-5) contains supplementary material, which is available to authorized users.

## Background

One of the promising directions in solving global problems of alternative energy and environment is the application of advanced technologies based on photocatalytic processes. Catalytically active TiO_2_ has been a subject of considerable attention due to its optical properties, chemical stability, non-toxicity, and high photoactivity. Therefore, this type of material is widely used for decomposition and synthesis of a number of organic compounds [1]. Furthermore, photocatalytic TiO_2_ has already been implemented in different practical fields such as antiviral and antibacterial agent [2, 3], for the destruction of cancer cells [4, 5], decomposition of volatile organic compounds, and water splitting. TiO_2_ is essential in medical and dental research due to its favorable properties, i.e., biocompatibility and low reactivity [6]. It already finds application in the construction of dental implants and hollow drug-containing structures. For instance, TiO_2_ nanotubes are implemented in medicine for gradual drug release [7].

One of the most important actual applications of TiO_2_ lies in the field of renewable energy conversion and storage [8]. The overall trend of the world’s energy systems requires broader introduction of hydrogen energy. In particular, the development of new technologies for hydrogen production through the design of suitable catalytic materials such as TiO_2_ is highly important. Advanced photocatalytic methods can lead to a significant cost reduction of both the hydrogen and the auxiliary systems. However, development of competitive materials with improved catalytic performance is required.

In particular, the doping of TiO_2_ structures with extra elements allows sensitizing the material in the visible wavelengths and/or increasing the photoactivity in the ultraviolet spectrum [9]. Doping with transition metals such as Fe, Mn, V, and Cr [10–15] displays low photocatalytic activity due to the thermal instability of the material [16] and furthermore creates environmental concerns. In the recent years, modification of TiO_2_ with nonmetal elements has received much attention, since they demonstrate effective doping, do not introduce ecological issues, and offer a low production cost. For example, the incorporation of nitrogen [17–22], carbon [23–26], sulfur [27–29], and iodine [30] in TiO_2_ structure can lower its band gap and therefore shift its optical response to the visible light region.

Among the other non-metallic elements sulfur doping displays a significant scientific interest and practical importance for the enhancement of TiO_2_ photocatalytic properties [31, 32].

It was recently found that sulfur-doped TiO_2_ exhibits a strong antibacterial effect under visible light, enabling effective inhibition of *Micrococcus lylae* and most of the Gram-positive bacteria [9]. The formation of hydroxyl radicals during irradiation of the structure by visible light plays a central role for the bactericidal activity of the material.

In most of the cases, the doping by appropriate amount of sulfur allows the use of S-TiO_2_ photoelectrodes in the visible spectrum region. Nevertheless, the structural aspects of sulfur integration in TiO_2_ are still debatable. Practically, the sulfur amount and its distribution in the TiO_2_ structure are to great extent influenced by the synthesis conditions. Recent studies in the field showed that sulfur is included in form of S^4+^ and S^6+^ predominantly on the surface of TiO_2_ nanoparticles [31]. On the other hand, there are significant proofs that sulfur is integrated in the TiO_2_ lattice forming S–Ti–O bonds [32].

The doping of TiO_2_ with carbon brings considerable potential advantages over other types of non-metallic doping. Carbon increases the conductivity of the structure [33], can accept the photon-excited electrons, enhances the separation of photo-generated carriers [34, 35], and displays visible light absorption in the wavelength of 400–800 nm [36, 37]. Most of the structural analyses of carbon-doped TiO_2_ indicated incorporation of this element into the lattice, substituting an O atom and forming O–Ti–C bond. The visible light absorbance in this case can be explained by the formation of a hybrid orbital above the valence band of TiO_2_ caused by carbon integration [38, 39].

Optimization and tuning the photocatalytic properties of the doped TiO_2_ is important with respect of its practical application. One of the appropriate methods in this direction is TiO_2_ co- and multiple doping, showing in a number of cases visible synergetic effects. Literature survey on sulfur-carbon TiO_2_ codoping shows that the topic is just approached and requires further analytical information on the influence of synthesis method and different structural factors.

In the present work, one-step TiO_2_ nanoparticles synthesis based on the interaction between thiourea and metatitanic acid is applied for sulfur and carbon anatase codoping. The integration of the doping elements is characterized by a number of physical methods, revealing the structural aspects of the doping process. A central task in our study is to test the photocatalytic activity of the SC-codoped TiO_2_ during decomposition of methylene blue, gas-phase photocatalytic oxidation of ethanol, and photocatalytic hydrogen generation from ethanol. According to the authors’ knowledge, the SC-TiO_2_ material has not yet been tested for the processes of photocatalytic ethanol oxidation and hydrogen generation. The impact of sulfur as a doping element in TiO_2_ is discussed in terms of its photocatalytic activity, compared to other already known TiO_2_ dopants.

## Methods

### Chemicals and Materials

Thiourea was provided by Wako Pure Chemical Industry. Other chemicals were obtained from commercial sources as guaranteed reagents and were used without further purification.

### Synthesis of SC-codoped and Non-doped TiO_2_ Nanopowders

Two samples of SC-codoped TiO_2_ nanopowders were synthesized using a solid-phase method; 12.2 g (*sample 1*) and 18.6 g (*sample 2*) of a metatitanic acid and 12.8 g (*sample 1*) and 6.4 g (*sample 2*) of thiourea were triturated in an agate mortar to obtain a homogeneous mass, which was further annealed in air atmosphere at 500 °C for 1 h.

The resulting powder samples differ in the amounts of doping elements. According to EDX analysis, sample 1 contains 0.23 wt.% sulfur and 10.68 wt.% carbon and sample 2 contains 0.45 wt.% sulfur and 2.38 wt.% carbon. The white non-doped TiO_2_ nanopowder was synthesized from metatitanic acid in the absence of thiourea under identical thermal conditions.

### Structural and Spectroscopic Characterization

The morphology and particle size of prepared nanoparticles were analyzed by SEM and TEM. A high-resolution scanning electron microscope Hitachi S-4800 II was used. The JEOL-JEM-1011 TEM microscope, operated at an accelerating voltage of 80 kV with a resolution of 0.2 nm, was used. The powder samples were prepared by air-drying a drop of a sonicated suspension onto copper grids.

The phase identification of sulfur-doped TiO_2_ structure was carried out by powder X-ray, using a Siemens D5000 diffractometer in reflection mode with Cu target Kα radiation. XRD patterns were recorded in the 2*θ* range of [15; 100°] with a step size of 0.02° and a stay time of 1 s/step.

Differential thermal analysis and thermogravimetric analysis (DTA-TG) was performed in air in the temperature range *T* = [20 °C; 800 °C] at a scan rate of 10 °C min^−1^ using derivatograph Q-1500 Paulik.

The optical properties were characterized by light absorption in the UV-vis and infrared region (FTIR) and Raman spectroscopy. The UV-vis absorption spectra for pure TiO_2_ and TiO_2_ doped by sulfur samples were recorded by an Evolution 300 UV-vis spectrophotometer (Thermo Scientific) in the range from 300 to 800 nm. The FTIR spectroscopy measurements were performed by Specord M-80 spectrometer in the middle infrared wavelengths (400–4000 cm^−1^). The Raman spectra were obtained using the Smart Raman DXR (Thermo Scientific) spectrometer. The semiconductor laser of 12-mW power and 780-nm wavelength was used as a light source.

The analysis of surface composition was carried out by means of X-ray photoelectron spectroscopy (XPS) using XSAM-800 Kratos spectrometer. The sample surface composition was determined using photoelectron line area ratio taking into account their sensitivity factors. The thickness of analyzed layer was ~5 nm.

The zeta potential measurement was performed by means of Zetasizer (Malvern Instrument Ltd.). The tested samples were suspended in water (pH = 7), vortexed and sonicated before measurement. The experiments were repeated three times for each sample, at *T* = 25 °C (±0.1 °C) and 1 mL of sample volume. The electrophoretic mobility and zeta potential were determined according to Smoluchowski’s equation.

EPR spectra of nanopowders were investigated in air at room temperature using spectrometer RADIOPAN X-85.

### Photocatalytic Measurements, Sample Preparation

Photocatalytic properties of synthesized powders were evaluated during the degradation of organic dyes (I), photocatalytic hydrogen generation in water-alcohol suspensions (II), and gas-phase oxidation of volatile organic compounds (IІI).

(I) Photocatalytic activity of gel samples containing SC-TiO_2_ nanoparticles has been tested for the decomposition of rhodamine B and methylene blue. Activation process has been performed using power LEDs with emission bands in the visible range of the spectrum (*λ* = 455 nm, *λ* = 525 nm).

Gelatin and polyvinyl alcohol (PVA) were selected as available and environment-friendly raw materials for obtaining gel—SC-TiO_2_ coatings. However, the application of gelatin is not practical due to the insufficient gel stability at low temperatures (3–6 °C). For the synthesis of gels based on PVA, aqueous solutions of varying content (5, 10, 15, 20, and 25 wt.%) were prepared. In order to dissolve PVA, the solutions were heated at 100 °C for about 10 min. Solutions containing 25 and 20 % (*w*/*w*) PVA have been instantly transformed into a gel, 15 % in 10–15 min and 5 % in 1 day. Therefore, 10 % PVA solution was selected for our research, since its gel transition takes approximately 2 h, which is sufficient for the necessary operations. The required amount of synthesized SC-TiO_2_ and hydrogen peroxide was added to the viscous PVA solution (~1 h after preparation) and stirred. This solution was applied to the inner surface of a quartz cuvette. After the formation of the gel, the surface is ready for photocatalytic reactions.

(II) Kinetic studies of hydrogen generation have been carried out in a thermostatted glass reactor (volume of 10.0 ml). The suspension of water, alcohol (ethanol containing 4 mol/L H_2_O), and sample of SC-TiO_2_ (0.05 g) was placed in the reactor. Irradiation of the reactor has been performed by a mercury lamp with DRSh ~ 1000 *λ* = 365 nm (UFS-2, the light intensity (*I*_0_ = 7 · 10^−6^ Einstein/min) was measured by ferrioxalate actinometer); 0.01 g of Pd/SiO_2_ was acting as a catalyst. The temperature of the reaction mixture was equal to 40 °C. During the reaction, the solution was not stirred. Solutions were evacuated before radiation. The amount of formed hydrogen in the reactor during irradiation of the solution was determined chromatographically by means of LHhM-8MD chromatograph with thermal conductivity detector. The measurements were performed by using carrier gas argon and chromatograph columns filled with molecular sieve NaX.

(III) The study of ethanol gas-phase oxidation kinetics was carried out in a thermostatted glass reactor (volume of 130 ml). Two milliliters of ethanol with a sample of SC-TiO_2_ (0.05 g) was placed in the reactor. Irradiation of the reactors has been performed by a mercury lamp with DRSh ~ 1000 *λ* = 310–390 nm (UFS-2); the light intensity was 1.2 · 10^−5^ Einstein/min and the light of mercury lamp with DRSh ~ 1000 *λ* ≥ 420–440 nm (UFS-2). The temperature of the reaction mixture was ~20 °C. During the reaction, the solution was not stirred. The amount of the ethanol remaining in the reactor and quantity of the generated acetaldehyde were determined chromatographically. The concentration of ethanol and acetaldehyde was measured by Chrome-5 chromatograph with flame ionization detector (column filled by SEPARON-SDA08 and carrier gas argon).

## Results and Discussion

### Synthesis and Structural Characterization

The processes taking place during the synthesis of SC-codoped TiO_2_ nanoparticles were characterized by means of thermogravimetry, where the thermal behavior of the individual reaction components (thiourea and metatitanic acid) and their mixture was analyzed.

**Fig. 1:**
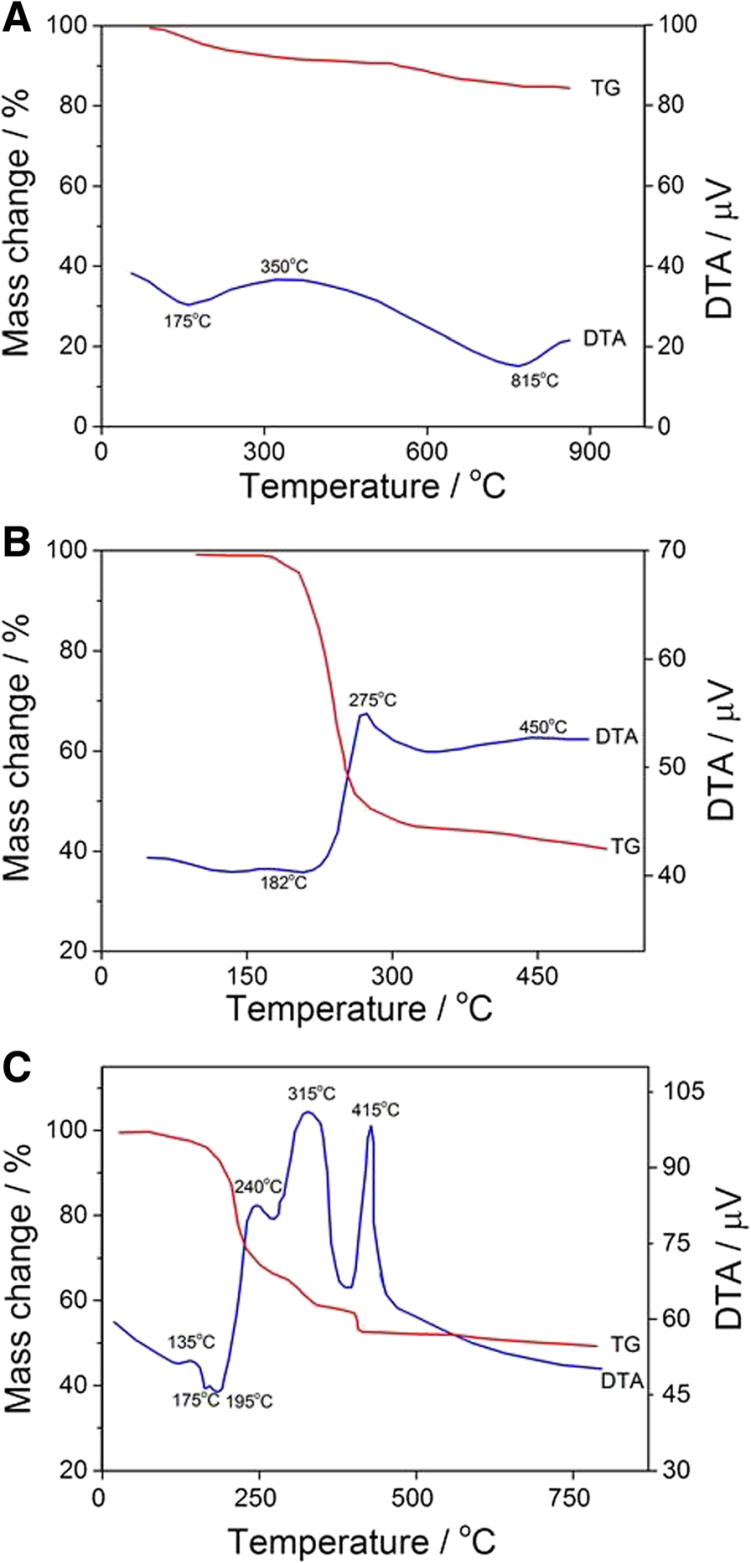
Thermogravimetric (DTA-TG) measurements for metatitanic acid (**a**), thiourea (**b**), and a mixture of thiourea and metatitanic acid (**c**)

According to the DTA (Fig. 1a), the thermolysis of metatitanic acid can be divided into three stages. The first step (100–205 °C), accompanied by an endothermic effect at 175 °C, corresponds to the dehydration of metatitanic acid. The second most time-consuming step is the crystallization of anatase, which takes place in the temperature range 205–815 °C. Finally, phase transformation in a rutile polymorph modification occurs at temperatures above 815 °C (third step). The total weight loss in the temperature range 0–500 °C is 9.4 wt.%.

The DTA data for the thermal behavior of thiourea are limited. The results of thermolysis of thiourea can be described using the reference data [40]. According to the latest, thiourea is melting at a temperature of 182 °C without loss of mass, accompanied by endothermic effect. Above this temperature, it decomposes forming NH_3_, H_2_S, CS_2_, and other gaseous products with significant weight loss. The endothermic effect related to thiourea melting was confirmed by the DTA measurements (Fig. 1b). The thermal analysis of thiourea shows further exothermic reaction above 275 °C caused by the decomposition of the compound and the process is accompanied by evolution of the above mentioned gaseous products. Finally, at temperatures higher than 450 °C, an exothermic process related to thermal oxidation of organic residues, including sulfur, which is evidently formed by decomposition of thiourea (b.p. sulfur = 444.6 °C) can be observed. Figure 1c shows the DTA curve of a mixture between metatitanic acid and thiourea. Based on the above described analysis of thermolysis of metatitanic acid and thiourea, the thermal decomposition of their mixture can be interpreted as follows:

(i) Endothermal process with a maximum at 135 °C related to a dehydration metatitanic acid;
(ii) Endothermal effects with a maximum at 175 and 195 °C connected to the melting of thiourea;
(iii) Exothermal process at 240 °C linked to crystallization of anatase within presence of thiourea and simultaneous thermal degradation to the above described gaseous products;
(iv) Exothermal processes with a maximum at 315 and 415 °C showing the thermal oxidation of the organic residues, sulfur, and continued crystallization of anatase.

X-ray phase analysis of the mixture between metatitanic acid and thiourea, annealed at 315 °C at a heating rate of 10 °C/min, displays the anatase crystallization, confirming our statements.

Based on the literature data [41, 42] and DTA results for the mixture of thiourea and metatitanic acid (Fig. 1c), it can be concluded that the loss of weight on heating is completed at a temperature of 415 °C, in contrast to weight loss during individual thiourea thermolysis, which lasts up to 500 °C and beyond. Obviously, in this case, the formed elemental sulfur oxidizes slowly; and in the case of the mixture, sulfur interacts with anatase. Thus, it was determined that the optimum temperature for the sintering of samples is 500 °C which is the temperature of completion of the process with an exothermic maximum at 415 °C.

**Fig. 2:**
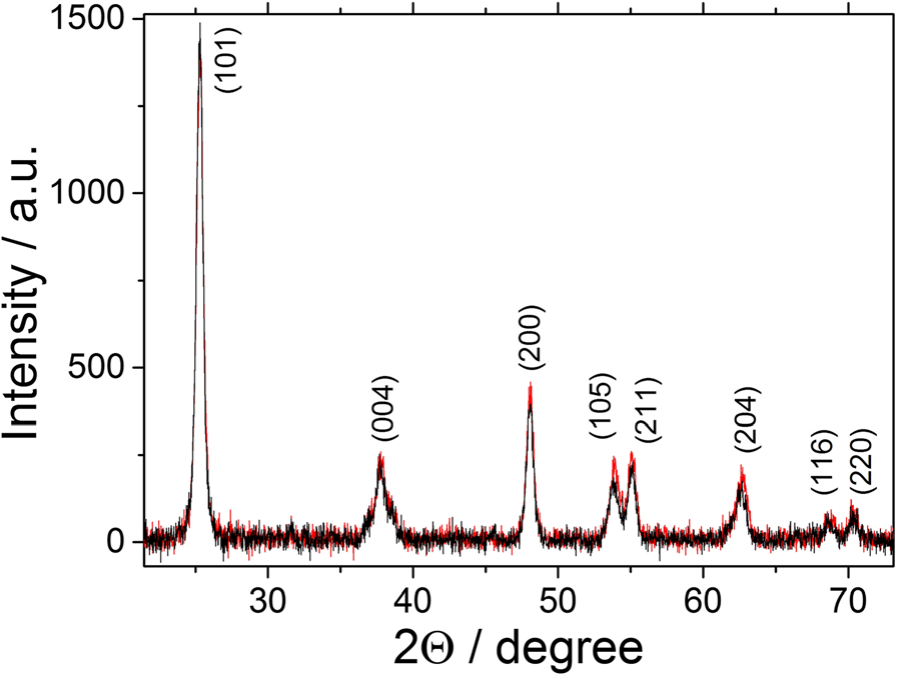
XRD pattern of doped SC-TiO_2_ powder materials. Sample 1—*black*, sample 2—*red*

The phase composition, obtained after thermal treatment of the material, was analyzed by XRD. The XRD patterns of doped SC-TiO_2_ nanoparticles are presented in Fig. 2.

Table 1 shows the values of the cell unit parameters for powders of pure TiO_2_ and TiO_2_, doped by sulfur and carbon, refined by the Rietveld method [43]. X-ray analysis for both samples displayed the existence of only one crystalline phase—the tetragonal modification of TiO_2_—anatase. Nevertheless, a slight *c* lattice parameter and volume increase due to incorporation of additional elements have been observed for the doped samples.

**Table 1:** TiO_2_ crystal lattice parameters assessed by Rietveld refinement method

Sample	Lattice parameters			
	a, Å	c, Å	c/a	V, Å
Pure TiO_2_	3.786	9.505	2.5105	136.25
Sample 1	3.785	9.527	2.5167	136.52
Sample 2	3.783	9.513	2.5145	136.17

**Fig. 3:**
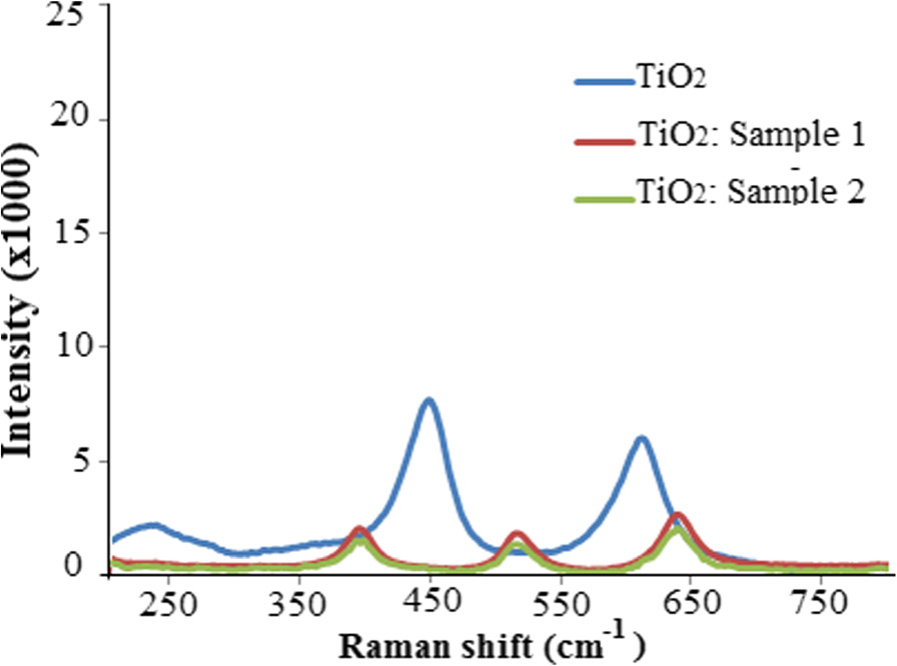
Raman spectra of non-doped TiO_2_ (*blue*) and SC-doped TiO_2_ powders (sample 1 *red*, sample 2 *green*)

The obtained Raman spectroscopy results confirmed the anatase crystal structure of doped TiO_2_ nanoparticles. Figure 3 shows Raman spectra of pure TiO_2_ and TiO_2_ doped by sulfur.

Spectra of sample I and sample II SC-doped TiO_2_ powders are similar with main lines at 638, 516, 396, and 145 cm^−1^, which confirm the structure of anatase. The Raman lines can be assigned as the E_g_, A_1g_, B_1g_, and E_g_ modes of the anatase phase, respectively [44]. The most informative is E_g_ band at 145 cm^−1^ arising from the external vibration of the anatase structure. If the line becomes weak or broad, there occur probably the local lattice imperfections [45].

**Fig. 4:**
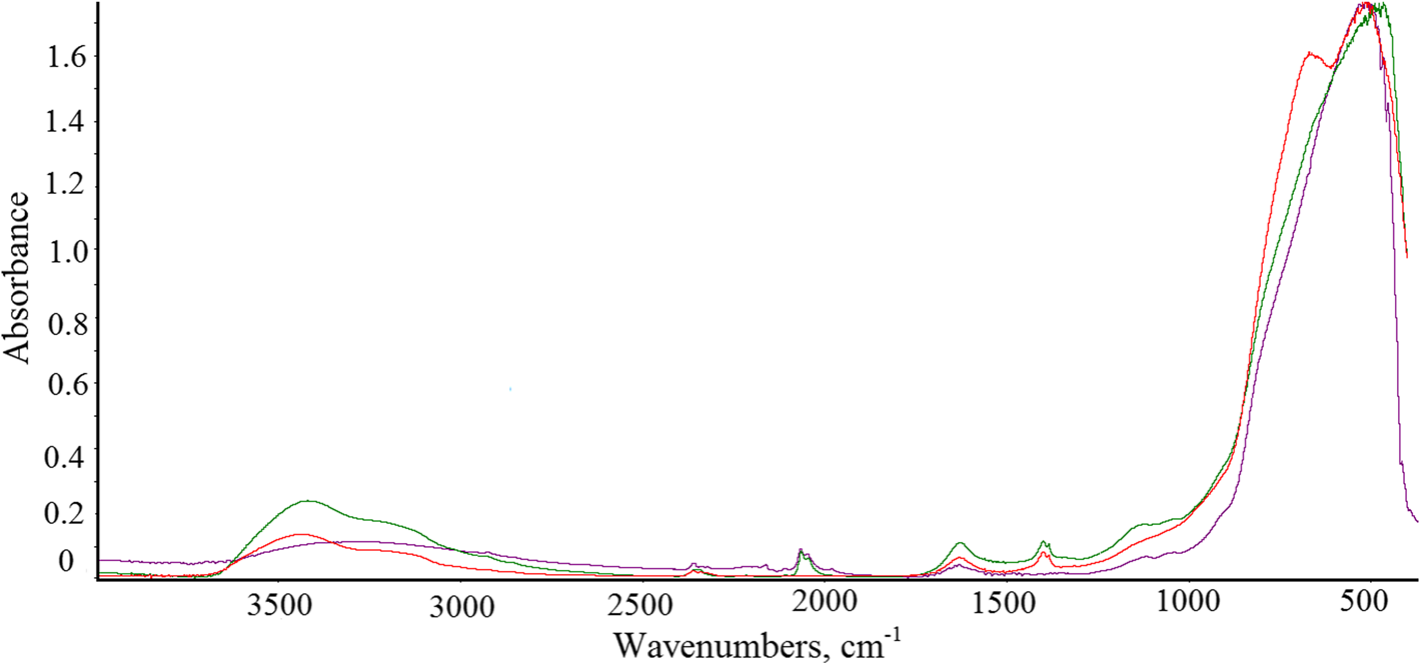
Infrared spectroscopy of TiO_2_ materials. Sample 1 *red*, sample 2 *green*, non-doped TiO_2_ *black*

The chemical structure of the synthesized materials has been further analyzed by IR spectroscopy (Fig. 4). Powder IR spectra allowed selecting six main absorption bands at 3700–3500, 2350–2000, 1650–1000, 1470–1180, 1180–1030, and 750–400 cm^−1^.

In the IR spectrum of the samples, the absorption band at 3700–3500 cm^−1^ is due to vibrations of OH bonds on the surface of TiO_2_. It can be seen that the absorption has a low intensity, due to a small amount of OH groups as a result of relatively high annealing temperatures [46]. The bands observed in the region at 2350–2000 and 1650–1500 cm^−1^ are related to a presence of adsorbed carbon monoxide [47]. The absorption at 1700–1000 cm^−1^ is characterized with vibrations in the surface-linked carboxyl compounds. The observed oscillations at 1560 and 1350–1420 cm^−1^ allowed us to attribute the resulting carbonate-carboxylate group to the bidentate carbonate [47–49]. Absorption band at 2200–2000 cm^−1^ relates to vibrations of the C=O bonds in complexes that are decomposed at 150 °C. Such form occurs only due to adsorption interaction of carbon (II) oxide at 20 °C with the surface of TiO_2_. The oscillation maxima at 2347, 2064, and 2215 cm^−1^ are related to CO adsorption, showing that the sample substantially adsorbs CO.

The band at 1470–1000 cm^−1^, visible only for the doped TiO_2_ samples, characterizes sulfur-containing functional groups. In particular, according to [48], the absorption at 900–700 cm^−1^ is associated with the vibrations of ν S–O. Furthermore, the presence of additional band in the range 1100–1040 cm^−1^ is linked to ν S=O vibrations and 1400–1310 cm^−1^ and 1230–1120 cm^−1^ to ν SO_2_ vibrations. In the particular case of SC-TiO_2_, the presence of absorption with maximums at 1379, 1348, and 1113 cm^−1^ can be attributed to variation of ν SO_2_ oscillations, characteristic for a sulfate group. The weak absorption peaks at 1260–1150, 1080–1010, and 700–600 cm^−1^ are representative for SO_2_OH group.

Vibrational frequency of ν O–O bonds in O^2−^ ions can be identified in the range 1180–1060 cm^−1^ [47]. As it can be seen from the figure, the peaks at 1113 and 1024 cm^−1^ are slightly shifted to lower frequencies but obviously are responsible for the vibrations of ν O–O. Actually, the existence of such bonds suggests activity of the synthesized powders towards photocatalytic oxidation processes.

Finally, the absorption at 750–400 cm^−1^ can be attributed to oscillations of the atoms in Ti–O and Ti–O–Ti bonds. In particular, the disappearance of the absorption band (750–550 cm^−1^) is due to the breaking of surface Ti–O bonds during high-temperature treatment [50].

**Fig. 5:**
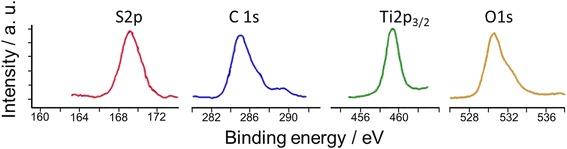
X-ray S2p, C1s, Ti2p_3/2_, and O1s core level photoelectron spectra for SC-TiO_2_ material

In order to characterize the chemical composition of the TiO_2_ structure and to determine the elemental oxidation states, the material was analyzed by means of XPS. X-ray core level photoelectron spectra for C1s, O1s, S2p, and Ti2p_3/2_ are presented in Fig. 5.

The analysis showed identical features for both types of doped SC-TiO_2_ samples. The results display big amount (about 30 %) of carbon, where three signal components at binding energies of 285.0, 287.0, and 289.0 eV are observed. The main maximum at 285.0 eV is attributed to elemental disordered carbon. However, multiple carbon sources, including adventitious carbon (in vacuo or external post-synthesis contamination) and chemical doping during synthesis theoretically can be the origin of its presence. Typically, the in vacuo contamination rates are very low compared with the detected carbon amount in our work. In most of the cases, adventitious carbon is in form of up to a nanometer thin island-type deposit, which thickness is far below the penetration depth of XPS source. Therefore, the detected high amount of surface carbon originates probably not from in vacuo contamination. Furthermore, the powder samples for XPS measurements are coated on a carbon tape attached to the sample holder, where a complete coverage of the substrate was achieved in order to exclude eventual signal from the carbon tape. On the other hand after annealing at high temperature in oxygen-containing atmosphere (for our experimental conditions 1 h at 500 °C in air), the existence of high amount of elemental carbon is less probable, suggesting that the high-intensity peak at 285 eV can be a result from external post-synthesis (not in vacuo) contamination.

The shoulder at 287.0 and the maximum at 289.0 eV are attributed to C–O and C=O bonds, where carbon can substitute lattice Ti atoms forming a Ti–O–C structure [51]. C=O groups are usually adsorbed on the surface, terminating the carbon interface or adsorbed on TiO_2_. Furthermore, the results from the infrared spectroscopy correlate well with XPS analysis confirming the presence of carbon-oxygen groups on the SC-TiO_2_ surface.

Oxygen is involved in the form of oxides (Eb = 530.6 eV), carbon-containing and OH groups adsorbed on the surface (shoulder at 532 eV). The binding energy of titanium (459.0 ± 0.3 eV) and sulfur (169.0 ± 0.3 eV) core shell for SC-TiO_2_ powders corresponds to established data for TiO_2_ and MeSO_4_ compounds, respectively. In particular, sulfur is found exclusively in the oxidation state +6.

In order to characterize the spatial distribution of the doping elements, XPS data were compared with EDX analysis, providing the elemental composition from powder volume. Information about the surface and volume elemental quantities in SC-TiO_2_ material is summarized in Table 2. It can be seen that sulfur and carbon are concentrated predominantly in the upper (periphery) layer of the nanoparticle. The quantification of the chemical elements detected by XPS analysis displays 2.8 % sulfur and 30.3 % carbon on the TiO_2_ nanoparticle surface, while the concentration of both elements drops to 0.45 % for S and 2.38 % for C detected in the powder volume. The spectra and detailed analytical results from EDX analysis can be seen in Additional file 1.

**Table 2:** Quantitative elemental analysis of TiO_2_ samples

Location	C, % at.	O, % at.	Ti, % at.	S, % at.
Surface (XPS)	30.3 C–H ~ 90 % C–O ~ 10 %	50.0	16.8 Е_b_ = 458.8 еV Ti^4+^ ~ 100 %	2.8 Е_b_ = 168,8 еV S^6+^ ~ 100 %
Volume (EDX)	2.38	40.16	57.02	0.45

**Fig. 6:**
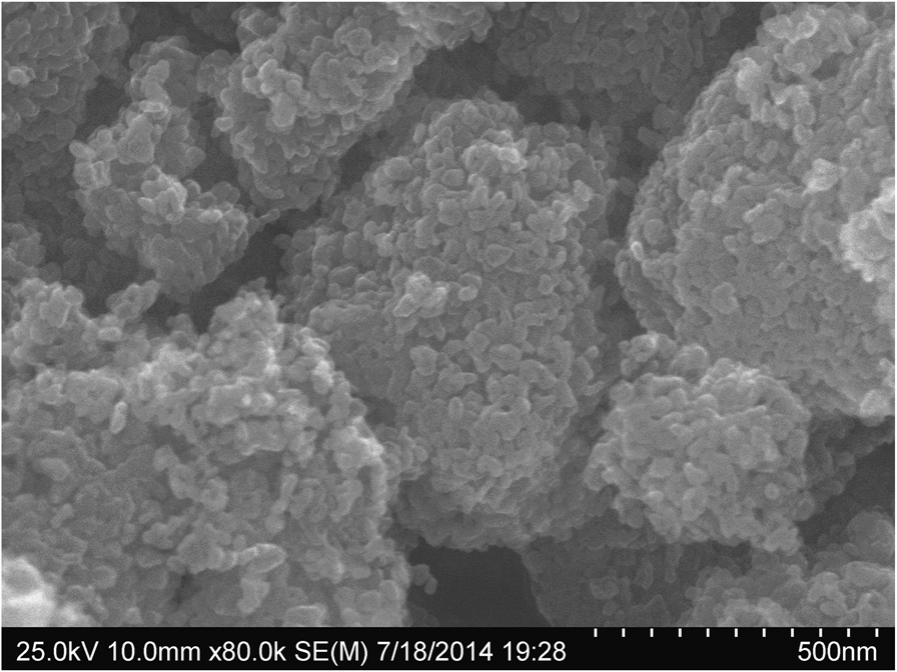
SEM imaging of the synthesized SC-TiO_2_ nanoparticles

Surface morphology of the synthesized TiO_2_ powder was characterized by SEM and TEM. SEM imaging showed micrometer-sized randomly distributed crystal aggregates, in the range of 5–15 μm (Fig. 6). The high magnification imaging revealed that the observed crystal aggregates consists of many 15–40-nm TiO_2_ nanoparticles.

**Fig. 7:**
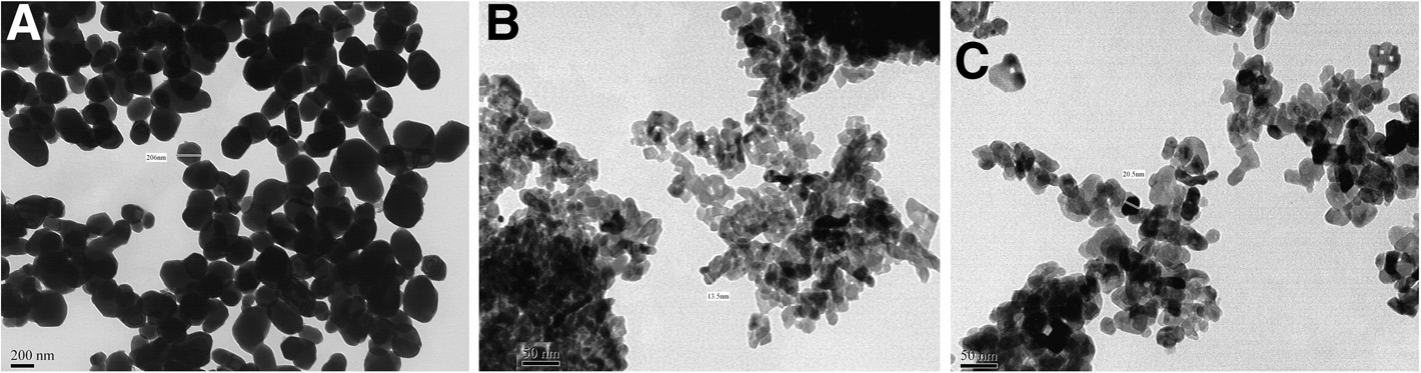
TEM imaging of SC-TiO_2_ nanopowder samples. TiO_2_ (**a**), sample 2 (**b**), sample 1 (**c**)

The obtained results by TEM particle morphologies are shown in Fig. 7. The size of pure TiO_2_ particles is under 200 nm. The particles in SC-TiO_2_ samples are about 10 times smaller. For the doped material, the range of separate nanoparticle size is from 10 to 30 nm, wherein an average size of particles for sample 1 is about 20 nm and for sample 2 is 15 nm.

Particle size and its distribution were assessed also by a laser dynamic light scattering (DLS) and the results complemented by Zeta-potential measurements are present in Table 3. The DLS particle size showed much larger dimensions in comparison to the obtained by microscopic methods. This suggests that nanoparticles, especially after doping by sulfur have a tendency to interact and to form bigger aggregates, which are not detected like separate nanoparticles but as aggregates. This effect was observed also on SEM images at low magnification (Fig. 6).

**Table 3:** The zeta potential, electrophoretic mobility, and dynamic light scattering analysis (hydrodynamic diameter and polydispersity) for non-doped TiO_2_ and SC-TiO_2_ powders

Sample	Zeta potential (mV)	Electrophoretic mobility (μmcm/Vs)	Particle size (nm)	Polydispersity index
Pure TiO_2_	−43.5 mV ±0.7	−3.18	411	0.24
Sample 1	−24.2 mV ±1.0	−1.75	683	0.42
Sample 2	−25.0 mV ±0.5	−1.67	660	0.46

The zeta potentials of all TiO_2_ samples, summarized in Table 3, showed negative values. The values of zeta potential for SC-doped TiO_2_ samples are about −25 mV which closely resemble the threshold value, indicating moderate stability of tested materials and low interaction affinity. Completely stable suspensions are considered, those with particles less than −30 or higher than 30-mV zeta potential. This condition is met by pure TiO_2_ and it is consistent with literature data [52].

**Fig. 8:**
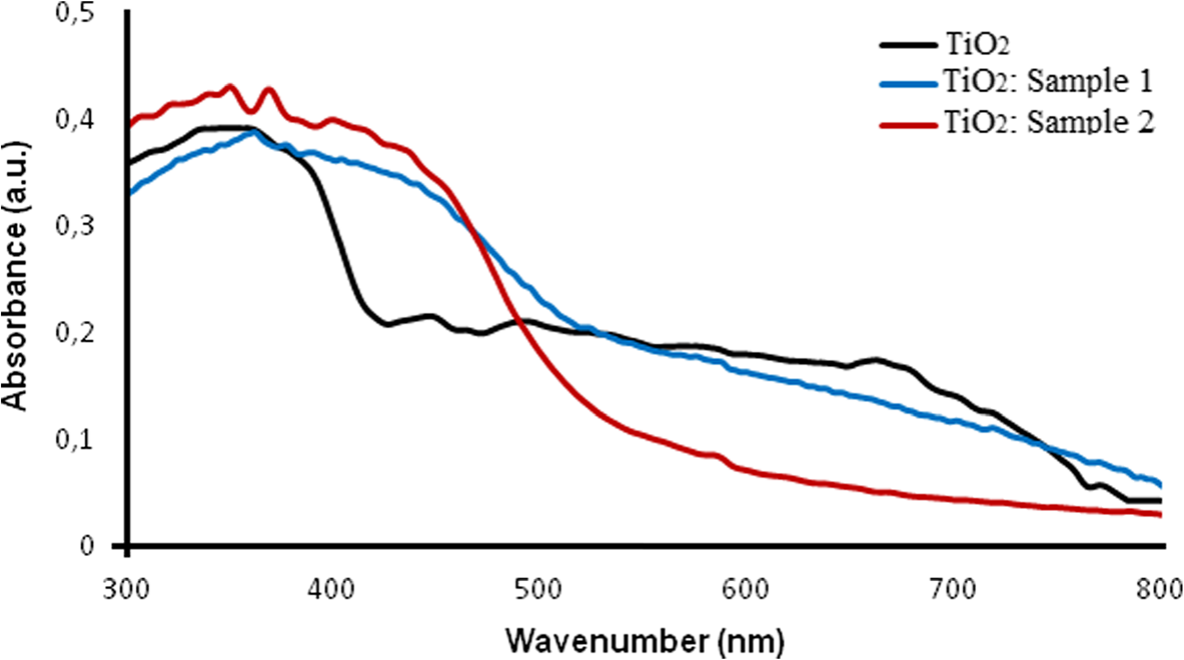
UV-vis absorption spectra of TiO_2_ and SC-doped TiO_2_ samples

Figure 8 shows UV-vis absorption spectra of TiO_2_ and SC-doped TiO_2_ samples. The maximum of absorbance for non-doped TiO_2_ was observed at around 350 nm, with the absorption edge around 400 nm. The edge defined by extrapolation the curve steep slope was about 415 nm. The SC-TiO_2_ samples showed broader absorption bands than non-doped TiO_2_ and marked shift of the absorption edge to a longer wavelengths. Both types of doped nanoparticle samples have similar absorption spectra in the UV and visible region. However, in case of sample 2, the intensity difference between the absorption band and the background was less than in the case of sample 1. The absorption edge for SC-doped nanoparticles is localized around 485 and 475 nm for sample 1 and sample 2, respectively (without the curve extrapolation). The observed absorption band broadening and the spectral shift to the visible region are signs for improvement of the photocatalytic properties of SC-TiO_2_ nanoparticles compared to standard TiO_2_. This suggests that the material is still catalytically active in UV region and additionally in some part of the visible spectrum [53].

**Fig. 9:**
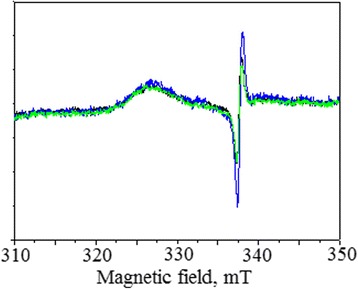
Electron paramagnetic resonance of the SC-TiO_2_ measured in the dark (*black*), under illumination by blue diode (*blue*) and under illumination by green diode (*green*)

EPR spectra of the powder samples, measured in dark and under illumination with blue (460 nm) and green (525 nm) diodes (0.01 W/cm^2^), are shown in Fig. 9.

It can be seen that the spectrum displays only one narrow line with the g-factor of 2.004 + −0.001. The amplitude of the line does not depend on the illumination in this spectral range. The line with g-factor 2.004 is associated with a single-electron-trapped oxygen vacancy [54]. Therefore, sensitization process of the nanopowders doped by sulfur and carbon is a result of the formation of additional oxygen vacancies in TiO_2_ structure.

### Photocatalytic Properties of SC-doped TiO_2_ Samples

Photocatalytic activity of the SC-doped TiO_2_ nanopowders was tested for the discoloration of solutions containing rhodamine B and methylene blue. The samples were further applied for photocatalytic generation of hydrogen in the visible light wavelength.

**Fig. 10:**
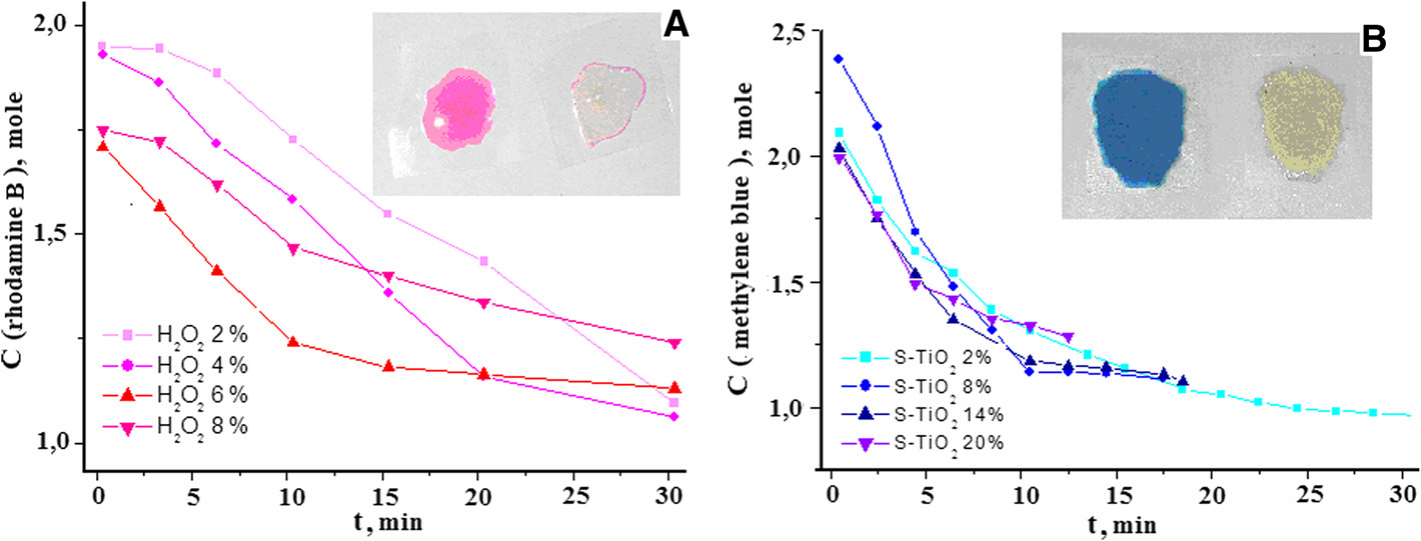
Dissociation of rhodamine B (**a**) and methylene blue (**b**) at the SC-TiO_2_ nanopowder sample 2

The photochemical degradation of rhodamine B was conducted in the presence of hydrogen peroxide. In order to determine the optimal concentration of hydrogen peroxide for the photocatalytic degradation reaction, the aqueous solutions of PVA, containing different H_2_O_2_ amounts (2, 4, 6, and 8 wt.%), were used for the preparation of the photocatalytic coatings. Next, rhodamine B solution was transferred in the cuvette with gel-like coating. Measurements of the sample absorption were performed at different activation intervals (Fig. 10a). The obtained results showed a significant impact of the H_2_O_2_ concentration on the photodegradation rate. The most efficient degradation of rhodamine B (*t* = 10 min) was observed for the sample containing 6 % hydrogen peroxide. To visualize the completion of photocatalytic decomposition reaction of rhodamine B, optical imaging of the solution discoloration is presented as insets in Fig. 10.

Keeping constant the optimal concentration of hydrogen peroxide (6 wt.%), the gels with different content of SC-TiO_2_ (2, 8, 14, and 20 wt.%) were prepared to determine the effect of its amount on the photocatalytic activity of the coating. Methylene blue was poured in the cuvette with gel-like coating and the measurement of the sample absorption was carried out similar to the previous one (Fig. 10a). The kinetic dependences (Fig. 10b) show that 10 times increase of SC-TiO_2_ amount in the gel results in reduction of the decomposition duration of methylene blue already by about 6 times. Similarly, images of the solution before and after the discoloration are inserted in Fig. 10b.

The obtained results show that the nanosized SC-TiO_2_, introduced in gel-like matrix, has high photocatalytic activity for the degradation of organic dyes. This effect can be explained, by the sulfur-carbon doping of the TiO_2_ particles, providing extension of the light absorption to the visible range of the spectrum. Additionally, the use of SC-TiO_2_ with H_2_O_2_ makes possible to enhance the generation of OH radicals, which facilitates the kinetics of organic pollutants oxidation.

The SC-TiO_2_ material was further tested for the reactions of gas-phase oxidation of ethanol under visible light and photocatalytic hydrogen generation from ethanol under ultraviolet light. The results summarized in Table 4 show that quantum yield of hydrogen generated during UV light irradiation of the synthesized SC-TiO_2_ samples is similar to the known reference samples [4] and commercial TiO_2_ Degussa P25 [5]. It should be noted that during irradiation of the SC-TiO_2_ samples with visible light, emission of molecular hydrogen was not observed.

**Table 4:** Characteristics of TiO_2_ samples

Sample	S, m^2^/g	γ (Н_2_)^a^, UV	V (С_2_Н_5_ОН)^b^, *10^7^ mol/min, UV
Sample 1	52.3	0.15	1.4
Sample 2	55.7	0.20	3.5
ТіО_2_ [4]	141.0	0.20	-
Degussa P25 [5]	50.0	0.21	2.7

^a^Quantum efficiency of molecular hydrogen

^b^Gas-phase oxidation rate of ethanol

It was established that SC-TiO_2_ samples synthesized by both methods are active in the photocatalytic oxidation of ethanol under visible light (sample 1 (C_2_H_5_OH) = 3.5 × 10^−8^ mol/min, sample 2 (C_2_H_5_OH) = 2.3 × 10^−8^ mol/min).

**Fig. 11:**
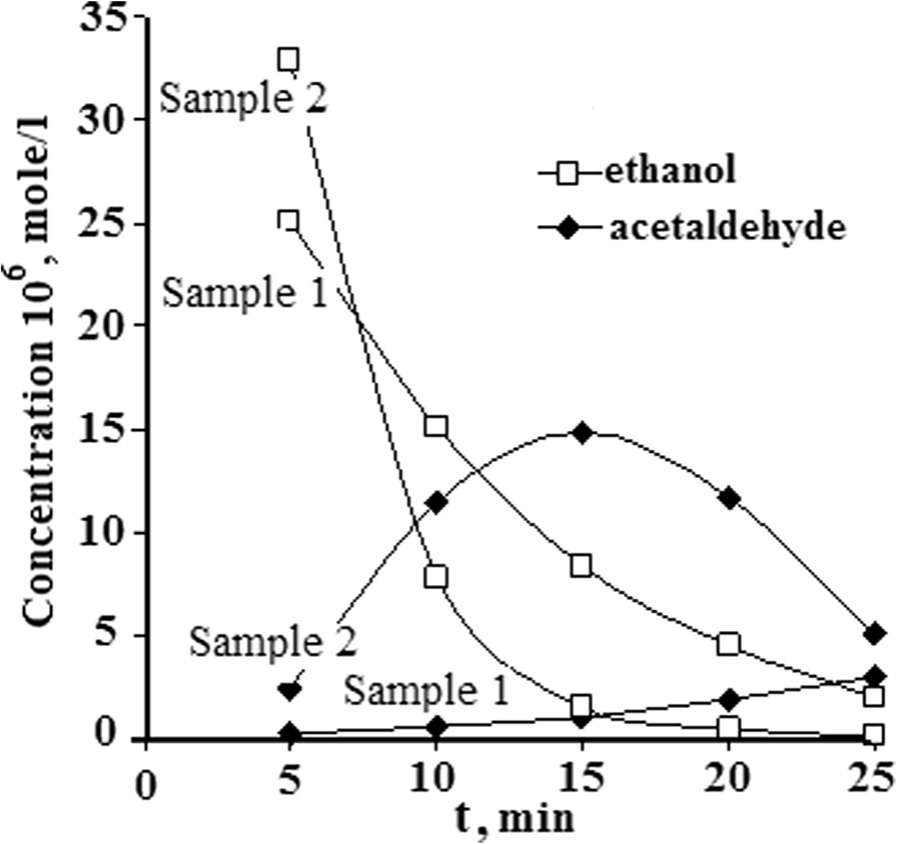
Kinetics of gas-phase oxidation of ethanol

The kinetic curves of gas-phase photocatalytic oxidation of ethanol by using sample 1 and sample 2 are presented on Fig. 11.

The results indicate that the ethanol oxidation kinetic depends strongly on the method for synthesis. In the case of sol-gel-synthesized sample (sample 2), the ethanol concentration rapidly drops in 15 min, which allows detecting the maximum of acetaldehyde generation at the moment of almost complete dissociation of ethanol. In contrast, ethanol oxidation catalyzed by TiO_2_ synthesized by solid-state method (sample 1) is characterized by lower reaction rate, where the complete conversion of ethanol is achieved for more than 25 min and no maximum of acetaldehyde concentration is observed. The differences in the catalytic activities of the samples can be explained by the particular surface chemistry and defect density achieved by both synthetic methods. In general, SC-TiO_2_ materials display good photocatalytic activity for the gas-phase ethanol oxidation, which makes them appropriate for air decontamination.

## Conclusions

Sulfur and carbon TiO_2_ codoping was accomplished by the interaction between thiourea and metatitanic acid. The nanopowder material was characterized by a number of physical methods.

The synthesis of the SC-TiO_2_ was studied by means of DTA-TG, which allowed determining the optimal temperature conditions of 500 °C for the synthesis and thermal post-treatment of the samples. Electron microscopy showed micrometer-sized (5–15 μm) randomly distributed crystal aggregates, consisting of many 15–40-nm TiO_2_ nanoparticles.

All types of TiO_2_ materials are crystallized in the anatase tetragonal phase. The incorporation of doping elements into the anatase crystal lattice was evidenced by XRD. It was confirmed that carbon is present in elemental form and also exists in carbon-oxygen compounds. The binding energy of titanium and sulfur core shells for SC-TiO_2_ powders corresponds to established data for TiO_2_ and MeSO_4_ compounds, respectively. XPS analysis revealed that sulfur is exclusively in 6+ oxidation state. Comparison of XPS and EDX data suggests that the doping elements are concentrated predominantly at the particle surface.

The EPR analysis revealed that the sensitization process of the nanopowders doped by sulfur and carbon is a result of the formation of additional oxygen vacancies into TiO_2_ structure, which are essential for the photocatalytic activity of the material.

The synthesized SC-TiO_2_ nanopowder can be applied in the processes of photocatalytic organic dyes degradation, gas-phase oxidation of water-alcohol mixtures, and hydrogen generation from ethanol, where the material exhibits high photocatalytic activity.

## Additional File

Additional file 1: EDX spectrum and analytical results for elemental composition of samples 1 and 2. **Figure S1.** EDX spectrum and analytical results for elemental composition of Sample 1. **Figure S2.** EDX spectrum and analytical results for elemental composition of Sample 2. (DOCX 32 kb)

## References

[1] Tian G, Fu H, Jing L, Tian C (2008). Synthesis and photocatalytic activity of stable nanocrystalline TiO(2) with high crystallinity and large surface area. J Hazard Mater.

[2] Pham HN, McDowell T, Wilkins E, Hazard T, Environ J (1995). Photocatalytically-mediated disinfection of water using TiO_2_ as a catalyst and spore-forming Bacillus pumilus as a model. Sci Eng Health.

[3] Wills RW, Gray JT, Fedorka-Cray PJ, Yoon KJ, Ladely S, Zimmerman JJ (2000). Synergism between porcine reproductive and respiratory syndrome virus (PRRSV) and Salmonella choleraesuis in swine. Vet Med Sci.

[4] Cai R, Kubota Y, Shuin T, Sakai H, Hashimoto K, Fujishima A (1992). Induction of cytotoxity by photoexcited TiO_2_ particles. Cancer Res.

[5] Kubota Y, Shuin T, Kawasaki C, Hosaka M, Kitamura H, Cai R, Sakai H (1994). Photokilling of T-24 human bladder cancer cells with titanium dioxide. Br J Cancer.

[6] Bousdras VA, Sindet-Pedersen S, Cunningham JL, Blunn G, Petrie A, Naert IE, Jaecques S, Goodship AE (2007). Immediate functional loading of single-tooth TIO_2_ grit-blasted implant restorations: a controlled prospective study in a porcine model. Part I: Clinical outcome. Clin Implant Dent Relat Res.

[7] Shokuhfar T, Hamlekhan A, Chang JY, Choi CK, Sukotjo C, Friedrich C (2014). Biophysical evaluation of cells on nanotubular surfaces: the effects of atomic ordering and chemistry. Int J Nanomedicine.

[8] Weng Z, Guo H, Liu X, Wu S, Yeungcd KWK, Chu PK (2013). Nanostructured TiO_2_ for energy conversion and storage. RSC Adv.

[9] Yu JC, Ho W, Yu J, Yip H, Wong PK, Zhao J (2005). Efficient visible-light-induced photocatalytic disinfection on sulfur-doped nanocrystalline titania. Environ Sci Technol.

[10] Yamashita H, Ichihashi Y, Takeuchi M, Kishiguchi S, Anpo M (1999). Characterization of metal ion-implanted titanium oxide photocatalysts operating under visible light irradiation. J Synchrotron Radiat.

[11] Klosek S, Raftery D (2001). Visible light driven V-doped TiO_2_ photocatalyst and its photooxidation of ethanol. J Phys Chem B.

[12] Anpo M, Takeuchi M (2003). The design and development of highly reactive titanium dioxide photocatalysts operating under visible light irradiation. J Catal.

[13] Ghosh AK, Maruska HP (1977). Photoelectrolysis of water in sunlight with sensitized semiconductor electrodes. J Electrochem Soc.

[14] Choi WY, Termin A, Hoffmann MR (1994). Role of metal-ion dopants in quantum-sized TiO_2_—correlation between photoreactivity and charge-carrier recombination dynamics. J Phys Chem.

[15] Wang YQ, Zhang L, Cheng HM, Ma JM (2000). Low-temperature preparation and visible light photocatalytic activity of mesoporous carbon-doped crystalline TiO_2_. Chem J Chinese U.

[16] Zhang F, Zhao J, Shen T, Hidaka H, Pelizzetti E, Serpone N (1998). TiO_2_-assisted photodegradation of dye pollutants II. Adsorption and degradation kinetics of eosin in TiO_2_ dispersions under visible light irradiation. Appl Catal B Environ.

[17] Sakthivel S, Janczarek M, Kisch H (2004). Visible light activity and photoelectrochemical properties of nitrogen-doped TiO_2_. J Phys Chem B.

[18] Asashi R, Morikawa T, Ohwaki T, Aoki K, Taga Y (2001). Visible-light photocatalysis in nitrogen-doped titanium oxides. Science.

[19] Ren W, Ai Z, Jia F, Zhang L, Fan X, Zou Z (2007). Low-temperature preparation and visible light photocatalytic activity of mesoporous carbon-doped crystalline TiO_2_. Appl Catal Environ.

[20] Irie H, Watanabe Y, Hashimoto K (2003). Nitrogen-concentration dependence on photocatalytic activity of TiO_2_-xNx powders. J Phys Chem B.

[21] Burda C, Lou YB, Chen XB, Samia AC, Stout J, Gole JL (2003). Enhanced nitrogen doping in TiO_2_ nanoparticles. Nano Lett.

[22] Diwald O, Thompson TL, Zubkov T, Goralski EG, Walck SD, Yates JT (2004). Photochemical activity of nitrogen-doped rutile TiO_2_(110) in visible light. J Phys Chem B.

[23] Khan SUM, Al-shahry M, Ingler WB (2002). Efficient photochemical water splitting by a chemically modified n-TiO_2_. Science.

[24] Irie H, Watanabe Y, Hashimoto K (2003). Nitrogen-concentration dependence on photocatalytic activity of TiO_2_-xNx powders. Chem Lett.

[25] Sakthivel S, Kisch H (2003). Photocatalytic and photoelectrochemical properties of nitrogen-doped titanium dioxide. Chem Phys Chem.

[26] Choi Y, Umebayshi Y, Yoshikawa M (2004). Fabrication and characterization of C-doped anatase TiO2 photocatalysts. J Mater Sci.

[27] Umebayashi T, Yamaki T, Itoh H, Asai K (2002). Band gap narrowing of titanium dioxide by sulfur doping. Appl Phys Lett.

[28] Umebayashi T, Yamaki T, Tanaka S, Asai K (2003). Visible light-induced degradation of methylene blue on S-doped TiO_2_. Chem Lett.

[29] Ohno T, Mitsui T, Matsumura M (2003). Photocatalytic activity of S-doped TiO_2_ photocatalyst under visible light. Chem Lett.

[30] Hong XT, Wang ZP, Cai WM, Lu F, Zhang J, Yang YZ, Ma N, Liu YJ (2005). Visible light-activated nanoparticle photocatalyst of iodine-doped titanium dioxide. Chem Mater.

[31] Wei F, Ni L, Cui P (2008). Preparation and characterization of sulfur-doped TiO(2)/Ti photoelectrodes and their photoelectrocatalytic performance. J Hazard Mater.

[32] Liu G, Wang X, Chen Z, Cheng HM, Lu GQ (2007). Supercritical preparation of a highly active S-doped TiO2 photocatalyst for methylene blue mineralization. Environ Sci Technol.

[33] Woan K, Pyrgiotakis G, Sigmund W (2009). Photocatalytic carbon-nanotube-TiO_2_ composites. Adv Mater.

[34] Zhang G, Teng F, Wang Y, Zhang P, Gong C, Chen L, Zhao C, Xie E (2013). Preparation of carbon-TiO_2_ nanocomposites by a hydrothermal method and their enhanced photocatalytic activity. RSC Adv.

[35] Kongkanand A, Kamat PV (2007). Electron storage in single wall carbon nanotubes. Fermi level equilibration in semiconductor-SWCNT suspensions. ACS Nano.

[36] Lee S, Lee Y, Kim DH, Moon J (2013). Carbon-deposited TiO_2_ 3D inverse opal photocatalysts: visible-light photocatalytic activity and enhanced activity in a viscous solution ACS. Appl Mater Interf.

[37] He Z, Que W, He Y (2014). Enhanced photocatalytic performance of sensitized mesoporous TiO2 nanoparticles by carbon mesostructures. RSC Adv.

[38] Zhang P, Shao C, Zhang Z, Zhang M, Mu J, Guo Z, Liu Y (2011). A high impact peer reviewed journal publishing experimental and theoretical work across the breadth of nanoscience and nanotechnology. Nanoscale.

[39] Valentin C, Pacchioni G, Selloni A (2005). Theory of carbon doping of titanium dioxide. Chem Mater.

[40] Fieser M, Fieser L (1974). Reagents for organic synthesis.

[41] Lettmann C, Hildenbrand K, Kisch H, Macyk W, Maier W (2001). Visible light photodegradation of 4-chlorophenol with coke-containing titanium dioxide photocatalyst. Appl Catal B.

[42] Reddy M, Jose R, Teng T, Chowdari B, Ramakrishna S (2010). Preparation and electrochemical studies of electrospun TiO2 nanofibers and molten salt method nanoparticles. Electrochim Acta.

[43] Rietveld HM (1969). A profile refinement method for nuclear and magnetic structures. J Appl Cryst.

[44] Choi HC, Jung YM, Kim SB (2005). Size effects in the Raman spectra of TiO2 nanoparticles. Vib Spectrosc.

[45] Zhang WF, He YL, Zhang MS, Yin Z, Chen Q (2000). Raman scattering study on anatase TiO2 nanocrystals. J Phys D Appl Phys.

[46] Tsyganenko A, Filimonov V (1974). Study adsorption of ammonia on the surface of metal oxides by IR spectroscopy. Uspekhi Fotoniki.

[47] Davydov A (1984). IK-Spektroskopiya v Khimii Poverkhnosti Okislov.

[48] Nakamoto K (1991). Infrared and Raman spectra of inorganic and coordination compounds.

[49] Kiselyov V, Krylov O (1978). Adsorbtsyonnye Procesy na Poverhnosti Poluprovodnikov i Dielektrykov.

[50] Bezrodna T, Gavrilko T, Puchkovska G, Shimanovska V, Babkov L (2002). Surface interaction in metal-doped TiO2 anatase-benzophenone heterogeneous systems. Funct Mat.

[51] Lin X, Fu D, Hao L, Ding Z (2013). Synthesis and enhanced visible-light responsive of C, N, S-tridoped TiO2 hollow spheres. J Environ Sci.

[52] Suttiponparnit K, Jiang J, Sahu M, Suvachittanont S, Charinpanitkul T, Biswas P (2011). Role of surface area, primary particle size, and crystal phase on titanium dioxide nanoparticle dispersion properties. Nanoscale Res Lett.

[53] Rockafellow EM, Stewart LK, Jenks WS (2009). Is sulfur-doped TiO_2_ an effective visible light photocatalyst for remediation?. Appl Catal Environ.

[54] Wang Y, Feng C, Zhang M, Yang J, Zhang Z (2010). Enhanced visible light photocatalytic activity of N-doped TiO_2_ in relation to single-electron-trapped oxygen vacancy and doped-nitrogen. Appl Catal Environ.

