# Supplementary material for: Synthesis, Characterization, and Photocatalytic Properties of Sulfur- and Carbon-Codoped TiO2 Nanoparticles
Source: Nanoscale Res Lett. 2016 Mar 12;11:140. doi: 10.1186/s11671-016-1353-5 (PMC4788660; doi:10.1186/s11671-016-1353-5)
Supplement: Additional file 1: — EDX spectrum and analytical results for elemental composition of samples 1 and 2. Figure S1. EDX spectrum and analytical results for elemental composition of Sample 1. Figure S2. EDX spectrum and analytical results for elemental composition of Sample 2. (DOCX 32 kb) [file 11671_2016_1353_MOESM1_ESM.docx]

**Synthesis, characterization and photocatalytic properties of sulphur and carbon codoped TiO_2_ nanoparticles**

***(Supporting information)***

Fig. S1 EDX spectrum and analytical results for elemental composition of *Sample 1*


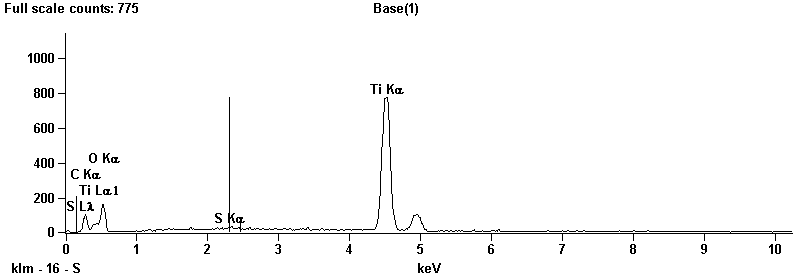


| ***Element***  ***Line*** | ***Weight %*** | ***Weight %***  ***Error*** | ***Atom %*** | ***Atom %***  ***Error*** |
| --- | --- | --- | --- | --- |
| ***C K*** | 10.68 | +/- 0.48 | 17.99 | +/- 0.80 |
| ***O K*** | 52.54 | +/- 1.59 | 66.43 | +/- 2.01 |
| ***S K*** | 0.23 | +/- 0.07 | 0.15 | +/- 0.05 |
| ***Ti K*** | 36.55 | +/- 0.47 | 15.44 | +/- 0.20 |
| ***Total*** | 100.00 |  | 100.00 |  |
|  |  |  |  |  |

Fig. S2 EDX spectrum and analytical results for elemental composition of Sample 2


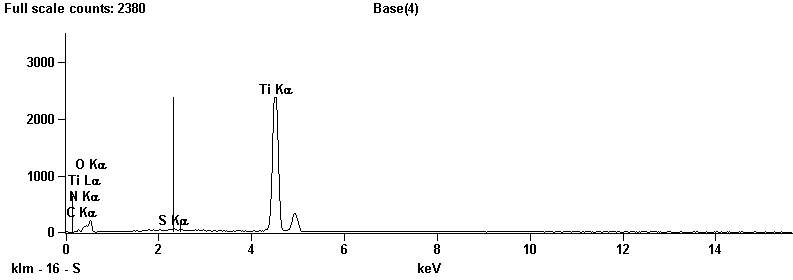


| ***Element***  ***Line*** | ***Weight %*** | ***Weight %***  ***Error*** | ***Atom %*** | ***Atom %***  ***Error*** |
| --- | --- | --- | --- | --- |
| ***C K*** | 2.38 | +/- 0.21 | 5.07 | +/- 0.45 |
| ***O K*** | 40.16 | +/- 1.21 | 64.15 | +/- 1.93 |
| ***S K*** | 0.45 | +/- 0.05 | 0.36 | +/- 0.04 |
| ***Ti K*** | 57.02 | +/- 0.43 | 30.43 | +/- 0.23 |
| ***Total*** | 100.00 |  | 100.00 |  |
